# Affective Modulation of Preparatory Cognitive Activity

**DOI:** 10.1002/pchj.70002

**Published:** 2025-03-04

**Authors:** Stefan Duschek, Antonio J. Sutil, Paulina Piwkowski, Thomas Rainer, Ulrich Ettinger

**Affiliations:** ^1^ Institute of Psychology Hall in Tirol Austria; ^2^ Department of Psychology University of Bonn Bonn Germany

**Keywords:** antisaccade, EEG, ERP, inhibition, response preparation

## Abstract

This EEG and eye‐tracking study investigated affective influences on cognitive preparation using a precued pro−/antisaccade task with emotional faces as cues. Negative information interfered with preparatory processes with high but not low executive function load.

Expectation of an upcoming event enables adjustment of sensory and motor systems to adequately respond to this event. Relevant processes include, for example, attentional activation, response selection, motor preparation or response inhibition, which are essential for optimising readiness to react. These processes vary according to characteristics of the prepared task and are supported by activity in lateral and dorsomedial prefrontal cortex (Connolly et al. 2002). Preparatory activity places high demands on cognitive resources, such that it may be particularly susceptible to interference by negative affect (Hoffmann et al. 2018).

This EEG study explored affective influences on basic and complex preparatory processes using a precued prosaccade/antisaccade task (Hutton and Ettinger 2006). In antisaccade trials, eye movements are recorded while participants are asked to look in the opposite direction of a visual stimulus (probe) that suddenly appears left or right of a central fixation point. In doing so, automatic behaviour, that is, a saccade towards the stimulus, must be inhibited in favour of an antisaccade requiring controlled action. Prosaccade trials that mainly demand automatic processes are typically used as control condition. Here, the probe was preceded by a visual cue, which instructed whether an antisaccade or a prosaccade had to be made in a trial. While a cue signalling a prosaccade should trigger basic preparatory attention, a cue indicating an antisaccade was expected to facilitate recruitment of processes with higher executive function load, particularly preparatory inhibition (Duschek et al. 2018). Affective influence was manipulated by using negative, neutral, and positive facial expressions as probes. During the task, event‐related potentials (ERPs) were recorded. Cortical activity during response preparation was indicated by the contingent negative variation (CNV) (Luck 2014). Processes of response execution were characterised by the N2 and P3a components, which arise after probe onset and are thought to relate to conflict processing and response inhibition (Polich 2007).

Thirty‐five university students (29 women, 6 men; age: *M* = 22.34 years, SD = 3.64) participated. At the beginning of each trial a small circle (cue) appeared for 1800 ms in the centre of the computer screen. Its colour informed the participant about the upcoming task condition (red for antisaccade, green for prosaccade). A picture of a face (probe) was subsequently presented left or right of the centre for 1000 ms. For antisaccades, participants had to look at the horizontal mirror image position of the probe; for prosaccades, they were required to look at the probe. The task comprised 6 blocks (40 trials each), stratified by the affective valence of the probes, with 2 blocks each of negative, neutral and positive probes (see Supporting Information for detailed task description).

An EyeLink 1000 (SR Research Ltd., Canada) was employed for video‐based eye‐tracking; data were processed using DataViewer software (SR Research). Performance parameters were saccade direction error rate and latency. Only directionally correct trials were included in the EEG analysis. The EEG signal was recorded using an actiCAP electrode system and actiCHamp amplifier (Brain Products Inc., Germany). BrainVision Analyzer software (Brain Products) was applied for data analysis. ERP amplitudes were computed for Cz (CNV), FCz (N2) and Pz (P3a) electrodes in the following time windows. CNV: 1600–1800 ms after cue onset; N2: 200–280 ms after probe onset, P3a: 260–400 ms after probe onset (see Supporting Information for details about data recording and processing). Two‐way ANOVAs were computed for performance parameters and ERP amplitudes, with the within‐subject factors Condition (antisaccade, prosaccade) and Valence (negative, neutral, positive).

Figure 1 displays the ERP findings. The ANOVA for the CNV revealed a main effect of Condition (F[1,34] = 16.49, *p* < 0.01, $\eta_{p}^{2}$ = 0.33), denoting larger CNV amplitude in antisaccade than prosaccade trials. Moreover, a Condition × Valence interaction arose (F[2,68] = 3.78, *p* = 0.028, $\eta_{p}^{2}$ = 0.10). Separate ANOVAs for each condition showed that the Valence effect was significant for antisaccade (F[2,68] = 4.21, *p* = 0.019, $\eta_{p}^{2}$ = 0.11) but not prosaccade trials. The CNV amplitude was smaller in antisaccade trials with negative probes than in those with positive (t[34] = 2.84, *p* < 0.01, *d* = 0.48) and neutral (t[34] = 1.89, *p* = 0.034, *d* = 0.32) probes. The ANOVAs for the N2 and P3a also showed a main effect of Condition (N2: F[1,34] = 39.91, *p* < 0.001, $\eta_{p}^{2}$ = 0.54; P3a: F[1,34] = 38.41, *p* < 0.001, $\eta_{p}^{2}$ = 0.53). N2 amplitudes were larger for antisaccade than prosaccade trials, and P3a amplitudes were larger for prosaccades than antisaccades. The ANOVAs for error rate and response latency revealed main effects of Condition (*ps* < 0.001). Error rate was higher, and latency was longer, in antisaccade than prosaccade trials (see Supporting Information for mean values of performance parameters).

**FIGURE 1:**
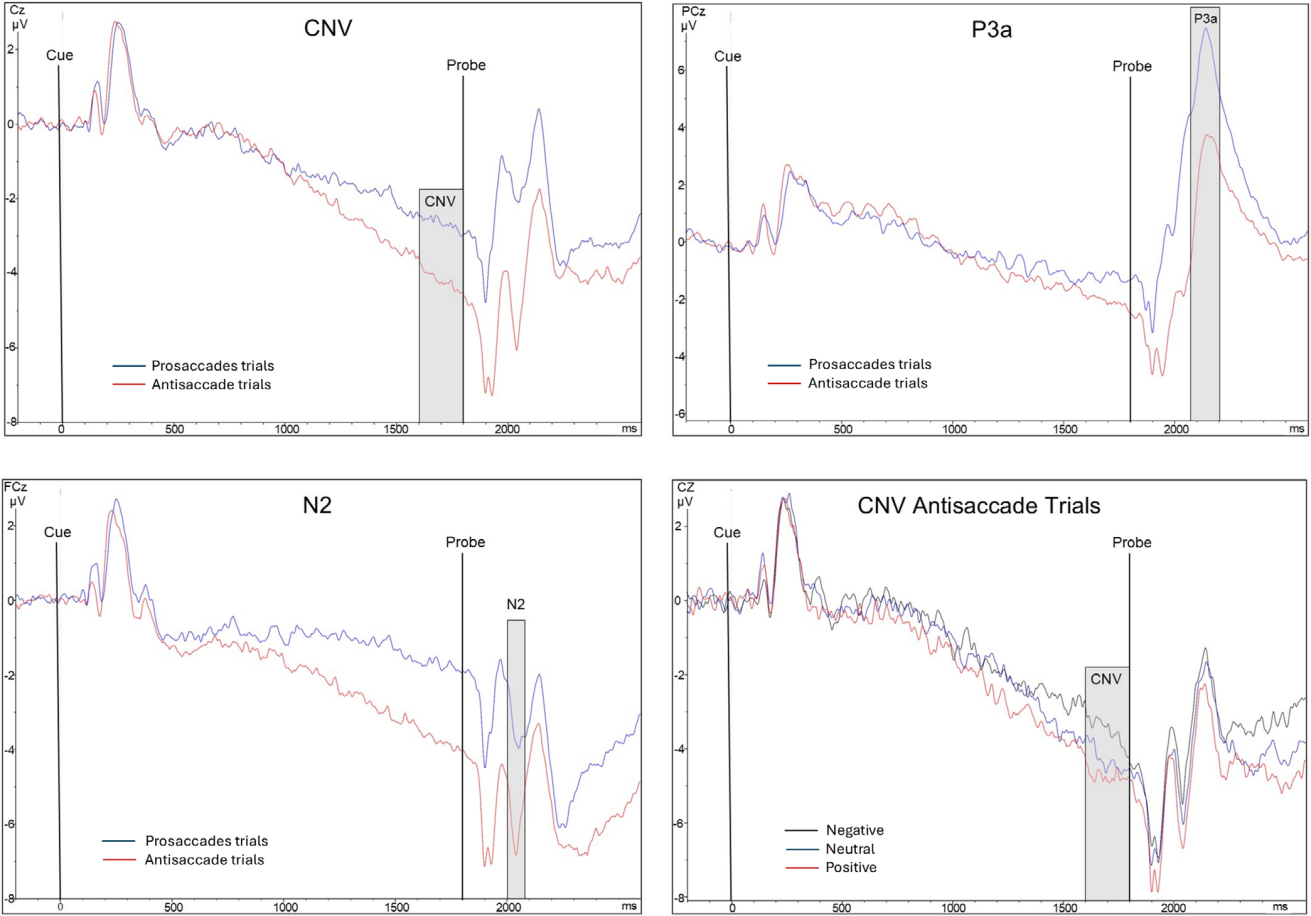
CNV (Cz), N2 (FCz) and P3a (Pz) for prosaccade and antisaccade trials; CNV (Cz) for antisaccade trials with negative, neutral and positive trials (grand averages).

The CNV indicates cortical resource mobilisation during preparation of a cognitive or motor response (Luck 2014). Its larger amplitude for antisaccade than prosaccades trials likely arose due to the higher preparatory demands of the more complex task condition. The probe N2 and P3a reflect response‐related cortical activity (Luck 2014). Greater resource allocation in the conflict between an automatic (prosaccade) and a controlled (antisaccade) response may account for the N2 amplitude difference between conditions. The P3a is associated with mismatch and conflict processing; in addition, its amplitude is inversely related to available processing capacity (Polich 2007). Therefore, the larger amplitude in the prosaccade trials than in the more complex antisaccade trials may relate to greater requirements on cognitive capacity by antisaccades.

The lower CNV amplitude for antisaccade trials with negative than those with neutral and positive probes reflects affective modulation of preparatory neural activity; preparation to respond in the context of negative information, as opposed to positive and neutral information, may require stronger neural resources (Cudo et al. 2018). The restriction of affective modulation to antisaccade preparation suggests stronger interference of negative affect with controlled processes (i.e., executive functions) than automatic stimulus‐driven processes (i.e., preparatory attention). Moreover, the lack of valence effects on the N2 and P3a supports the notion that negative affect can more strongly impair the preparation of a task than its actual execution (Hoffmann et al. 2018). The observations accord with previous studies showing diminished cortical blood flow during preparation of various cognitive tasks in depressed patients (c.f. Hoffmann et al. 2018).

While poorer behavioural performance in antisaccade than prosaccade trials accords with previous observations (c.f. Hutton and Ettinger 2006), performance was unaffected by stimulus valence. However, as the precued prosaccade/antisaccade task does not provide specific behavioural parameters of task preparation, different affective effects on task preparation and execution could not be investigated on the behavioural level. Furthermore, neural measures such as ERPs may be more sensitive to affective modulations than behavioural indices. Specifically, negative affective effects observed in the CNV may be masked at the behavioural level by compensatory strategies. Previous studies investigating affective modulation of the CNV revealed both positive and negative results (e.g., Cudo et al. 2018; Wouwe et al. 2011). However, the variation of negative affective impact on neural activity as a function of executive function load of preparatory processes is a novel finding of this study.

## Ethics Statement

The study was approved by the Board for Ethical Questions in Science of the University of Innsbruck (Austria). All participants provided informed consent.

## Conflicts of Interest

The authors declare no conflicts of interest.

## Supporting information


Data S1.

## Data Availability

The research data of the study are available to the public via the repository Open Science Framework (OSF, https://osf.io/nwhp3/).
