# Supplementary material for: Affective Modulation of Preparatory Cognitive Activity
Source: Psych J. 2025 Mar 4;14(3):463–5. doi: 10.1002/pchj.70002 (PMC12133230; doi:10.1002/pchj.70002)
Supplement: Supplementary file 1 — Data S1. [file PCHJ-14-463-s001.docx]

**Affective Modulation of Preparatory Cognitive Activity**

Duschek, S., Sutil, A.J., Piwkowski, P., Rainer, T. & Ettinger, U.

**Supplementary Material: Detailed description of the task, psychophysio-logical recordings and data processing; behavioral task results**

**A. Precued antisaccade task**

The task was presented using ExperimentBuilder software (version 2.4.193; SR Research Ltd., Ottawa, Canada). It was comprised of 6 blocks, stratified by the affective valence of the probe stimuli, i.e. negative, neutral or positive (two blocks for each valence). Angry, afraid and sad facial expressions served as negative probe stimuli, happy faces served as positive probe stimuli, and emotionless faces served as neutral probe stimuli (50% female and 50% male faces for each valence and in each block). Images were taken from the Karolinska Directed Emotional Faces Battery (Lundqvist et al., 1998)^1^. There were 40 trials per block (overall 240 trials); participants were informed about the valence of the probe stimuli before the beginning of each block. Expressions of the same affective valence were used within a block to increase affective effects across trials.

The screen background colour during the task was black. At the beginning of each trial the cue was shown in the centre of the screen for 1800ms, consisting of a red or green circle (diameter: visual angle 0.65°) with a black hole (diameter: visual angle: 0.17°) in the centre. Its colour informed the participant about the upcoming task condition (red for antisaccade, green for prosaccade). Subsequently, and without gap or overlap, the probe (height: visual angle 8.02°, width: visual angle 6.3°) was shown with the image centre at ±8.15° horizontally (50% left and 50% right in each block) from the centre of the screen for 1000ms. Antisaccade and prosaccade trials were equally frequent in each block. Between trials a white central fixation stimulus (identical size as cue) was shown for 1000-1400ms, jittered in 100ms steps around a mean of 1200ms. The sequence of the 6 blocks was randomized across subjects.

After the task, participants rated the probe stimuli individually on the valence and arousal dimensions using the Self-Assessment Manikin Scale (SAM; Bradley & Lang, 1994)^2^. The ratings confirmed the expected valence evaluations of the facial expressions (negative: M=2.71, SD=0.70; neutral: M=4.26, SD=0.72; positive: M=6.78, SD=0.93; all t[34]≥19.25, all p<.001). Arousal ratings were lower for neutral (M=3.29, SD=1.20) than negative (M=4.76, SD=1.44) and positive (M=4.89, SD=1.32) expressions (all t[34]≥5.58, all p<.001), but did not differ between positive and negative expressions (t[34]=0.63, p=.53).

^1^ Lundqvist, D., Flykt, A. & Ohman, A. (1998). The Karolinska Directed Emotional Faces (KDEF). Karolinska Institute: Department of Clinical Neuroscience, Psychology Section.

^2^ Bradley, M. M. & Lang, P. J. (1994). Measuring emotion: The Self-Assessment Manikin and the Semantic Differential. *Journal of Behavior Therapy and* *Experimental Psychiatry*, *25*, 49-59. https://doi.org/10.1016/0005-7916(94)90063-9

**
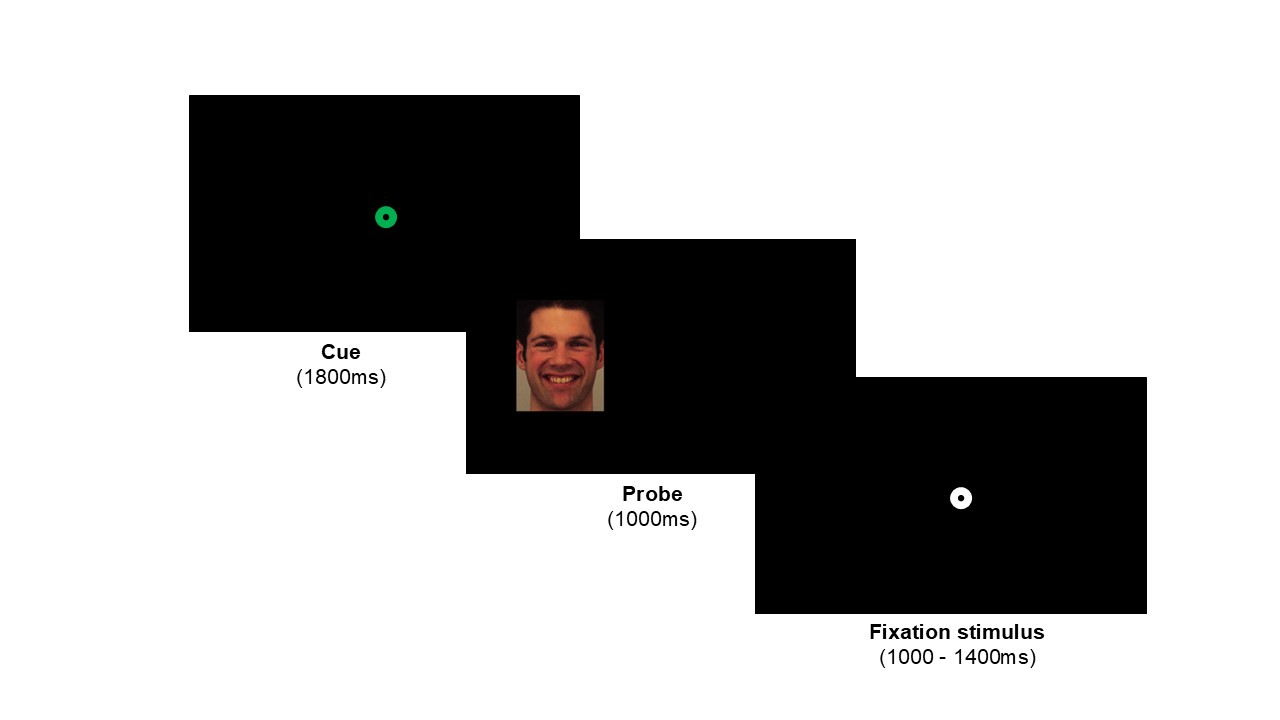
**

**Figure S1.** Scheme of the cued antisaccade task

**B. Data acquisition**

An EyeLink 1000 system (SR Research Ltd., Ottawa, Canada; Host Software, version 4.56) was applied for video-based dark pupil and corneal reflection eye tracking. Horizontal and vertical gaze positions of the right eye were recorded at a sampling rate of 500 Hz. Recordings were made in remote mode using a chin rest without further stabilization of the head at a distance of 90 cm from eyes to screen. The device has a spatial resolution of <0.1° RMS, an average accuracy of between 0.25° and 0.5° and an average end-to-end sample delay of <3ms (SD=1.11ms). The inbuilt EyeLink five-point horizontal and vertical calibration procedure was performed before the beginning of the task.

The EEG signal was taken from 64 scalp positions (10/10 system) using an active electrode system (actiCAP, Brain Products, Inc., Gilching, Germany) and an actiCHamp amplifier (Brain Products, Inc.). The EEG was recorded against Cz and re-referenced offline to the linked mastoids. A ground electrode was located at FPz. Electrode impedances were maintained below 25 kΩ. Signals were stored at a sampling rate of 1000 Hz.

**C. Data processing**

Oculographic data were processed using DataViewer software (version 4.3.210; SR Research Ltd.). Saccade detection criteria included minimum amplitude of 1° and minimum latency of 80ms. Trials with a blink after 100ms prior to probe onset or between probe onset and saccade were excluded. Trials were also excluded when the X coordinate of the saccade starting point differed by more than 2° from the centre of the screen. Performance measures were direction error rate and response latency. A direction error in an antisaccade trial was counted when the first valid saccade was made towards the peripheral probe, and in prosaccade trials when it was made in the opposite direction of the probe. Error rate was given by the ratio between errors and valid trials and converted to percent. Saccadic response latency was computed in milliseconds (ms) as the interval between probe onset and saccade onset for directionally correct saccades. Trials containing direction errors, blinks or saccades starting earlier than 80ms after probe onset were excluded from EEG analysis.

For offline analysis of EEG data, BrainVision Analyzer software (version 2.3, Brain Products, Inc.) was applied. The data were resampled at 500 Hz and digitally filtered (low-pass at 40 Hz, high-pass at 0.1 Hz, notch at 50 Hz). Eye movement and blink artifacts were corrected using independent component analysis. Data were segmented in epochs (from -200ms relative to cue onset to +800ms relative to probe onset), baseline corrected (from -200ms to 0ms relative to cue onset) and averaged across trials according to experimental condition (antisaccade, prosaccade) and valence (negative, neutral, positive). An artifact rejection protocol with the following criteria was applied. Maximal allowed voltage step/ms = 50 µV, minimal allowed amplitude = -100 µV, maximal allowed amplitude = 100 µV, maximal allowed absolute voltage difference within an epoch = 200 µV, and lowest allowed activity = 0.5 µV. Across all participants, on an average 3.24 trials (SD=3.41 trials) had to be rejected per condition due to these criteria. To select adequate time windows of the ERPs of interest, sequences were averaged across all electrodes, trials, and participants. The time windows defined according to this procedure were as follows. CNV: 1600-1800ms after cue onset; probe N2: 200-280ms after probe onset, probe P3a: 260-400ms after probe onset. N2 and P3a amplitudes were determined using global maxima detection in the corresponding time windows. As the CNV did not show a clearcut peak, the average voltage in the corresponding time window was computed for this component.

**D. Behavioural task results**

The results for error rate and response latency on the task are displayed in Figure S2.

**
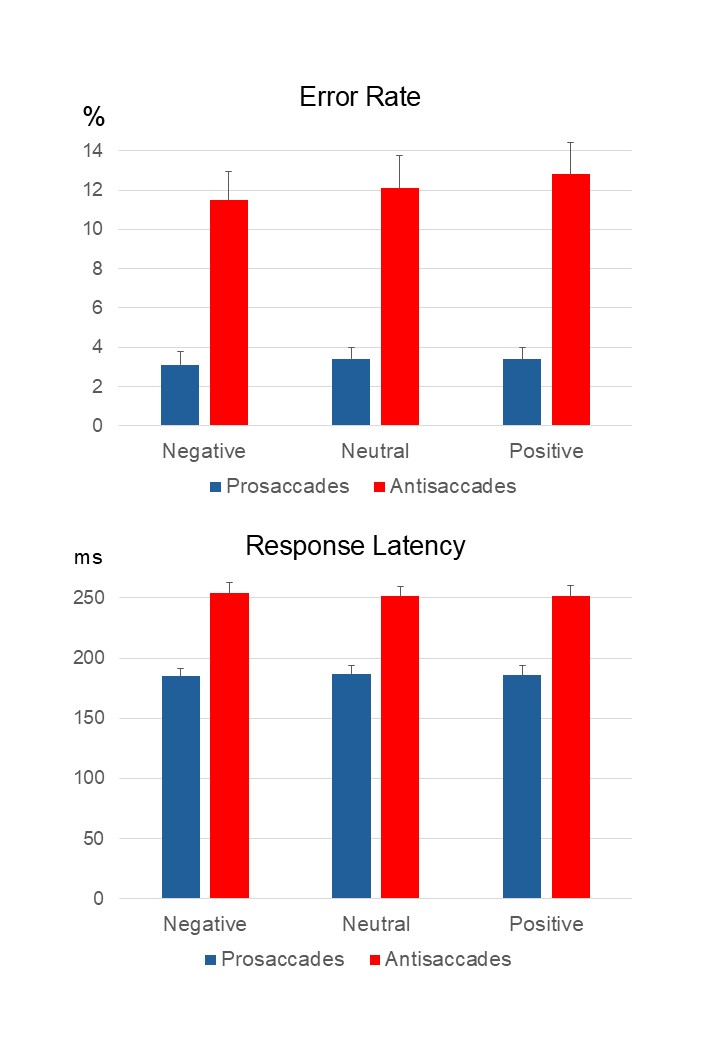
**

**Figure S2.** Results of the cued antisaccade task (bars represent standard errors of the mean)
